# The Influence of Decentration on Higher-Order Aberrations in Artisan Aphakic Intraocular Lens Implantation Eyes

**DOI:** 10.1155/2020/7601524

**Published:** 2020-04-09

**Authors:** Aizhu Miao, Min Zhang, Tianhui Chen, Yi Lu

**Affiliations:** ^1^Department of Ophthalmology and Vision Science, Eye and ENT Hospital of Fudan University, Shanghai, China; ^2^NHC Key Laboratory of Myopia (Fudan University), Key Laboratory of Myopia, Chinese Academy of Medical Sciences and Key Laboratory of Visual Impairment and Restoration of Shanghai, Shanghai, China

## Abstract

**Purpose:**

To characterize the influence of decentration on higher-order aberrations of Artisan aphakic intraocular lens implantation eyes. *Setting*. Department of Ophthalmology and Vision Science, Eye and ENT Hospital of Fudan University, Shanghai, China.

**Design:**

Retrospective case series.

**Methods:**

Twenty-three eyes of 18 patients were retrospectively examined. Location of the IOL was imaged using a slit-lamp, and decentration (the distance from the center of a pupil to the geometric center of the IOL) was measured using the AutoCAD 2007 software. Ocular and internal higher-order aberrations were measured using the wavefront analyzer KR-1W (Topcon) and their correlation with decentration were analyzed. Coma, spherical, 3rd-, 4th-order, trefoil, tetrafoil aberrations, ocular, and internal higher-order aberrations were calculated for a 4.0 mm pupil diameter.

**Results:**

The mean age of patients was 33.6 ± 21.4 years (ranging from 7 to 72 years). The mean follow-up period was 28.2 ± 10.5 months (ranging from 12 to 52 months). The mean postoperative best-corrected visual acuity (BCVA) was 0.19 ± 0.19 logMAR (range −0.18–0.52). The mean decentration was 0.57 ± 0.28 mm (range 0.15–1.21 mm). There was no correlation between Artisan IOL decentration and ocular higher-order aberrations, internal higher-order aberrations, coma, spherical, 3rd-, 4th-order, trefoil, and tetrafoil aberrations, respectively. There was a positive correlation between Artisan IOL decentration and 2nd-astig aberrations when 2nd-astig aberrations were less than 0.1.

**Conclusions:**

The position of Artisan IOLs showed slight decentration and the amounts of decentration were not large enough to influence higher-order aberrations.

## 1. Introduction

The Artisan intraocular lens (IOLs), one of the iris-fixated rigid intraocular lenses, can be implanted in both phakic and aphakic eyes. Since the initial iris-claw phakic IOL study for the correction of myopia in 1978 [1], a number of studies have been published demonstrating the safety and efficacy of the Artisan phakic IOL for correcting myopia, hyperopia, and astigmatism [2–6].

When capsular support is absent in cataract surgery, angle-supported or iris-fixated anterior chamber IOLs and trans-sclerally sutured or iris-fixated posterior chamber IOLs can be implanted. Angle-supported IOLs have not been widely used due to a high incidence of corneal endothelial cell loss. Although trans-sclerally sutured posterior chamber IOLs are more acceptable, they are associated with disadvantages such as longer surgical time and a high incidence of intraoperative and postoperative complications. The latter includes lens tilting, decentration, retinal detachment, vitreous hemorrhage, and suture erosion [7]. Artisan IOL is an alternative for aphakic eyes when capsular support is absent. The procedure is relatively simple and associated complications are fewer compared with angle-supported and trans-sclerally sutured IOLs.

Although numerous studies have reported the safety and efficacy of Artisan IOLs, few of them have investigated the effect of Artisan IOLs on vision quality after IOL implantation. Previous studies have shown that tilt and decentration of IOLs may influence vision quality, but experience in Artisan IOLs is scarce. Due to the fact that Artisan IOLs are fixed on the iris with clips on both sides of the optics and the optical surface of the IOL is almost completely parallel with the surface of the iris, it is less likely that the tilt of IOL is able to influence the higher-order aberrations. The purpose of the present study was to determine whether decentration of Artisan IOLs in aphakic eyes could influence higher-order aberrations.

## 2. Patients and Methods

### 2.1. Patient Selection and Study Design

This retrospective study investigated 23 eyes from 18 patients who had their first or second Artisan aphakic intraocular lens implantation in the Department of Ophthalmology, Eye and ENT Hospital of Fudan University, Shanghai, China. Patients who had best-corrected visual acuity better than logMAR 0.52 after surgery were included. Exclusion criteria were corneal, iris or pupil abnormalities, traumatic eyes, previous retinal detachment, previous inflammation of the anterior or posterior segment, and postoperative complications such as glaucoma and retinal abnormalities. The mean age of these patients and the mean follow-up period as well as other demographic information were collected. Decentration (the distance between the center of a pupil and the IOL) was measured using the AutoCAD 2007 software after completing the slit-lamp photography. Aberrations were measured using the Wavefront Analyzer KR-1W (Topcon), and their correlation with decentration was analyzed.

### 2.2. Surgery

All procedures were performed by the same surgeon (Dr. Lu Y). Under general or local anesthesia upon patients' demand, a 5.5 mm scleral tunnel incision was made 1 mm posterior to the limbus, and two paracentesis were performed at 10 and 2 o'clock positions. Then, 1% acetylcholine was injected to constrict the pupil and the anterior chamber was filled with an ophthalmic viscosurgical device, and an Artisan IOL was inserted into the anterior chamber, rotated with a hook to an appropriate position, and centred over the pupil. The iris was hooked between the claw-like footplates to achieve perfect IOL centration over the pupil. Then peripheral iridotomy was performed at the 12 o'clock position, and the viscoelastic material was removed. Finally, the incision was closed with three interrupted 10-0 nylon sutures.

### 2.3. Decentration Measurements

**Figure 1:**
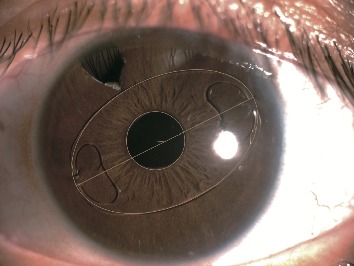
Decentration measurement using AutoCAD 2007 software on a silt-lamp photograph.

The Artisan aphakic intraocular lens is a PMMA IOL with a length of 8.5 mm and an optical zone of 5.0 mm. Decentration of the IOL referred to the distance between the center of a pupil and the IOL. Slit-lamp photographs were taken using a Nikon-6 camera (Nikon Corp, Tokyo, Japan)/EAS-1000 anterior eye segment analysis system (Nidek, Gamagori, Japan). Decentration was measured on slit-lamp photographs using the AutoCAD 2007 software shown in Figure 1. The diameter of the IOL (8.5 mm) was used as a reference for decentration measurements.

### 2.4. Aberration Assessment

All patients underwent a complete ophthalmic examination during the follow-up including slit-lamp biomicroscopy, best-corrected visual acuity (BCVA), and slit-lamp photography. The ocular and internal higher-order aberrations were measured using the wavefront analyzer KR-1W (Topcon, Tokyo, Japan) in a dark room with lights switched off. All measurements of KR-1W were taken by a single experienced technician. This device can measure the ocular and the corneal aberrations, which renders the calculation of the internal aberrations more accurately. The KR-1W provided a measured value of the 4 mm and 6 mm optical zones for aberrations. The pupil diameter of all patients in the KR-1W examination of the present study was larger than 4 mm; therefore, pupil dilation was not necessary before KR-1W examination, and we chose 4 mm aberration for analysis.

### 2.5. Statistical Analysis

Data were presented in the format of mean ± SD, and the correlation between aberrations and decentration was analyzed using Lowess curves.

## 3. Results

The average age of the 18 participants in the present study was 33.6 ± 21.4 years ranging from 7 to 72 years. Among them, 14 (77.8%) were men and 4 (22.2%) were women. The mean follow-up period after surgery was 28.2 ± 10.5 months, ranging from 12 to 52 months. The mean postoperative best-corrected visual acuity (BCVA) was 0.19 ± 0.19 logMAR, ranging from −0.18 to 0.52. The mean decentration of the IOL was 0.57 ± 0.28 mm, ranging from 0.15 to 1.21 mm. Figure 1 shows a slit-lamp photograph.

**Figure 2:**
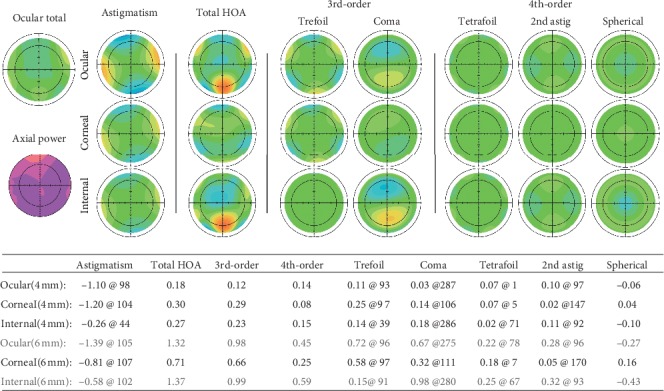
Aberration measurements using KR-1W. We analyzed aberrations with the 4 mm optical zone.

The AutoCAD 2007 software was used to find the center of the pupil and the center of the Artisan IOL. Decentration (the distance between the center of a pupil and the IOL) was measured with the IOL length (8.5 mm) as a reference.  Figure 2 shows aberration measurements using the KR-1W system. The diameter of all pupils in the present study was larger than 4 mm. Therefore, 4 mm aberration was selected for analysis. The ocular, corneal, and internal higher-order aberrations are shown in Figure 2. The 3rd-, 4th-order, trefoil, coma, tetrafoil, 2nd-astig, and spherical aberrations are also shown.

**Figure 3:**
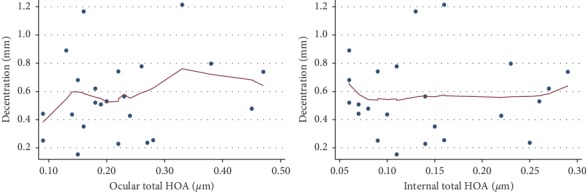
Relationship between ocular total higher-order aberrations (HOA), internal total higher-order aberrations (HOA), and decentration.

**Figure 4:**
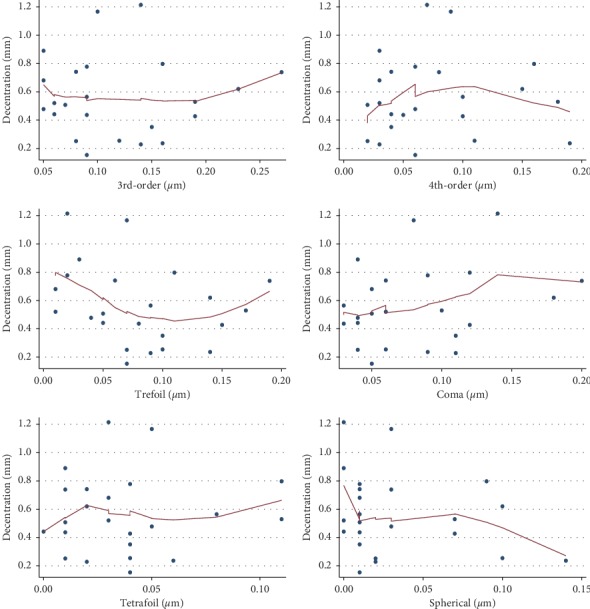
Relationship between 3rd-order, 4th-order, trefoil, coma, tetrafoil, spherical aberrations, and decentration.

**Figure 5:**
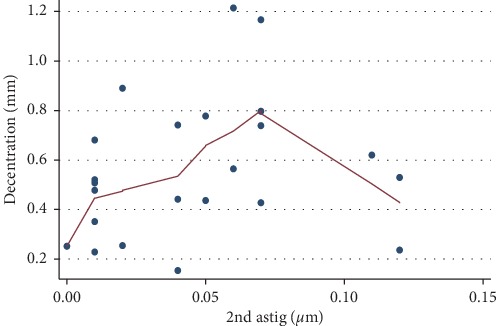
Relationship between 2nd-astig aberrations and decentration.

Lowess curves were used to study the relationship between aberrations and decentration. There was no correlation between ocular total higher-order aberrations, internal total higher-order aberrations, and decentration (Figure 3). No relationship between the 3rd-order, 4th-order, trefoil, coma, tetrafoil, spherical aberrations, and decentration was observed either (Figure 4). However, a positive correlation between 2nd-astig aberrations and decentration was observed when 2nd-astig aberrations were less than 0.1 (*P*=0.021) (Figure 5).

## 4. Discussion

Numerous studies have investigated the safety and efficacy of Artisan IOLs. However, the effect of Artisan IOLs on vision quality after IOL implantation remains unclear. By analyzing the relationship between higher-order aberrations and decentration after IOL implantation during the follow-up period, we found that there is no relationship between ocular total higher-order aberrations, internal total higher-order aberrations, the 3rd-order, 4th-order, trefoil, coma, tetrafoil, spherical aberrations, and decentration. Interestingly, we found a positive correlation between 2nd-astig aberrations and decentration when 2nd-astig aberrations were less than 0.1. These indicate that IOL implantation is not only safe but also unharmful to visual acuity and other higher functions.

Artisan aphakic IOL can be implanted when the capsule is inadequate. Studies have shown good clinical outcomes with this IOL. Wu et al. reported that the iris-claw intraocular lens implantation is a simpler procedure, which takes shorter time to learn than scleral-fixated posterior chamber intraocular lens implantation in correcting aphakia without sufficient capsular support [8]. The Artisan aphakic IOL has been reported safe to use for adults and children. Guell et al. evaluated 128 aphakic eyes with secondary iris-claw anterior chamber IOL implantation and found it an effective, predictable, and safe procedure in the first 5 years of follow-up [9]. Chen et al. reported satisfactory results of secondary anterior Artisan IOL implantation in 72 patients during a three-year follow-up [10]. Van Der Meulen et al. reported that Artisan IOL implantation in 13 aphakic patients led to few complications and good visual acuity after removing dislocated lens fragments with a pars plana vitrectomy [11]. There are also cases of Artisan aphakic IOL implantation in children with good clinical outcomes. Artisan aphakic IOL is both feasible and effective in managing ectopia lentis in children [12–14]. Sminia et al. also reported Artisan aphakic IOL implantation in children with traumatic pediatric cataract with a follow-up period of 11.0 years [15] and in children with partial aniridia caused by perforating ocular trauma for a follow-up period of 8.9 years [16]. Apart from anterior chamber implantation, Artisan aphakic IOL can also be successfully used for posterior iris fixation through a scleral tunnel incision [17–20]. Forlini et al. studied 320 eyes (141 post-traumatic eyes, 122 post-cataract eyes, and 57 eyes with penetrating keratoplasty). The mean post-operative best-corrected LogMAR visual acuity was 0.6, 0.3, and 0.6 for a mean follow-up period of 5.3 years. They concluded that retropupillary implantation of the Artisan iris-claw intraocular lens for secondary implantations is a valid alternative strategy [20]. Although the posterior position respects the anatomic features of the anterior segment and is safer for corneal endothelium, the procedure is more challenging and posterior complications are possibly more frequent compared with anterior chamber implantation.

However, few studies have investigated the effect of Artisan IOL on visual quality. Higher-order aberrations are considered important determinants of visual quality and widely used to evaluate visual quality following IOL implantation surgery. To our knowledge, little research has been conducted to study higher-order aberrations in Artisan phakic IOL eyes. Buhren et al. proposed that higher trefoil was due to the incision size and higher spherical aberration was related to Artisan phakic IOL implantation [21]. Tahzib et al. analyzed patient satisfaction after Artisan Phakic IOL implantation in 120 eyes which had myopia corrected. They analyzed the correlations between aberrations and night vision score, glare score, and night driving score through higher-order aberration measurements and questionnaires [6]. In another study, they found that postoperative spherical aberration and trefoil-y increased in the Artisan phakic IOL group compared to the Artiflex phakic IOL group [22]. Peris-Martinez et al. also found that the Artisan IOL was approximately 13% better than the Artiflex IOL in average modulation for myopia [23]. Chung et al. found a slight increase in high-order aberrations (HOAs) under photopic conditions and a decrease in contrast sensitivity (CS) for high myopia, both of which returned to baseline at 3 months [24]. Qasem et al. followed patients undergoing Artisan phakic IOL for myopia, hyperopia, or astigmatism for five years and showed an increase in total higher-order aberrations and spherical aberration [2].

Centration of the IOLs predominantly determines the visual quality after IOL implantation. A few methods have been used to measure decentration of IOLs, such as Purkinje imaging, Scheimpflug imaging, ultrasound biomicroscopy, anterior segment optical coherence tomography, and anterior segment photographic documentation [25]. In the present study, we used anterior eye segment analysis system to take photographs and to determine decentration of Artisan aphakic IOLs. The latter was determined as the deviation of the center of the IOL from the center of the pupil. Our method is similar to previous studies in measuring decentration of Artisan phakic IOLs for myopia correction [6].

The correlation between decentration and aberrations has been widely studied in model eyes and in living eyes. Different amounts of tolerable decentration for aberration correcting IOLs were reported using eye models. Holladay et al. showed a maximum decentration of 0.4 mm [26]. Piers et al. found a limit of 0.8 mm for decentration [27]. Altman et al. studied the theoretical optical performance of three IOL designs and found spherical and aberration-correction IOLs decentered 0.5- and 1 mm, respectively, and induced asymmetrical HOAs, whereas aberration-free IOLs were not affected by decentration [28]. Perez-Vives et al. compared the optical quality of implantable Collamer lens (ICL) with and without central hole at different degrees of decentering and found that coma aberration increased with ICL decentration, but the values were clinically negligible and did not have a significant effect on the simulated visual performance [29]. In another study, the optical quality of aspheric toric IOLs at different degrees of decentering was analyzed in vitro, and it was found that coma aberration increased with IOL decentration [30]. Baumeister et al. studied the impact of spherical and aspheric foldable IOL tilt and decentration on optical quality after cataract surgery. The mean optic decentration was 0.19 ± 0.12 mm for or the spherical IOL and 0.27 ± 0.16 mm for the aspheric IOL. Decentration did not significantly affect the best-corrected visual Strehl ratio of the optical transfer function, which was calculated from the wavefront aberration [31]. Suaer et al. investigated the position of IOLs implanted in the ciliary sulcus with a defective capsular bag during phacoemulsification. They found pronounced lens decentration but did not find a negative effect on total eye high-order aberrations, i.e., spherical aberration and coma [32].

To our knowledge, this is the first study on the correlation between higher-order aberrations and decentration in Artisan aphakic intraocular lens implantation eyes. Tahzib. et al. reported that the mean ± SD amount of decentration of the IOL was 0.36 ± 0.14 mm (range, 0.14–0.74 mm) in 120 Artisan phakic IOL implantation eyes for myopia. They found no correlation between higher-order aberrations and the amount of decentration but a positive correlation between the amount of IOL decentration and horizontal coma [6]. In another study, a mean amount of Artisan aphakic IOL decentration of 0.25 ± 0.12 mm (range 0.10–0.46 mm) was reported. No eye had decentration greater than 0.5 mm, and no significant correlation between decentration and postoperative aberrations was found [22]. In the present study, the wavefront analyzer KR-1W was used to measure the aberrations. The aberration of aphakic IOL implanted eyes dominantly come from the IOL and the cornea. The wavefront analyzer KR-1W allows to estimate the internal optical aberration of the eye, nearly corresponding to the IOL. Previous studies evaluating the aberrations and decentration in Artisan phakic IOL implanted eyes did not separately analyze internal aberrations and total aberrations of the eye. In our analysis, we found no correlation between Artisan IOL decentration and ocular higher-order aberrations and internal higher-order aberrations, consistent with previous studies in that decentration was not large enough to impair visual quality [33]. However, we did find a correlation between Artisan IOL decentration and 2nd-astig aberrations. Although a couple of studies showed the correlation between IOL decentration and astigmatism [34, 35], the correlation between decentration and 2nd-astig aberrations has not been reported. Korynta reported the correlation between IOL decentration and astigmatism when IOL tilt is >5 degrees and IOL decentration >1 mm [34]. In the other study, Atchison also demonstrated that tilting or decentration of IOL will adversely affect the quality of retinal images with the introduction of coma-like aberrations and astigmatism [35].

There are some limitations in the present study. First, the number of our cases was comparatively small. Second, we lack preoperative aberration measurements, which would enable us to evaluate changes of the aberrations before and after Artisan aphakic IOL implantation. Third, we did not use mydriatics before aberration measurements. Aberrations would change depending on different pupil diameters. Fourth, we did not evaluate patients' fixation stability in the present study. Fixation stability was correlated with macular sensitivity and BCVA [36], and it might influence slit-lamp photography and decentration measurements.

In conclusion, the position of Artisan aphakic IOLs in the present study showed slight decentration, and the amounts of decentration were not large enough to have negative impact on higher-order aberrations. However, there was a positive correlation between Artisan IOL decentration and 2nd-astig aberrations when 2nd-astig aberrations were less than 0.1.

## Data Availability

Some or all data, models, or code generated or used during the study are available from the corresponding author on request.

## References

[1] Fechner P. U., Van Der Heijde G. L., Worst J. G. F. (1989). The correction of myopia by lens implantation into phakic eyes. *American Journal of Ophthalmology*.

[2] Qasem Q., Kirwan C., O’Keefe M. (2010). 5-year prospective follow-up of artisan phakic intraocular lenses for the correction of myopia, hyperopia and astigmatism. *Ophthalmologica*.

[3] Güell J. L., Morral M., Gris O., Gaytan J., Sisquella M., Manero F. (2008). Five-year follow-up of 399 phakic artisan-verisyse implantation for myopia, hyperopia, and/or astigmatism. *Ophthalmology*.

[4] Venter J. (2009). Artisan phakic intraocular lens in patients with keratoconus. *Journal of Refractive Surgery*.

[5] Silva R. A., Jain A., Manche E. E. (2008). Prospective long-term evaluation of the efficacy, safety, and stability of the phakic intraocular lens for high myopia. *Archives of Ophthalmology*.

[6] Tahzib N. G., Bootsma S. J., Eggink F. A. G. J., Nuijts R. M. M. A. (2006). Functional outcome and patient satisfaction after artisan phakic intraocular lens implantation for the correction of myopia. *American Journal of Ophthalmology*.

[7] Bellamy J. P., Queguiner F., Salame N., Montard M. (2000). Secondary intraocular lens implantation: methods and complications. *Journal Français D’Ophtalmologie*.

[8] Wu J., Liang G. L., Zheng Q. Y. (2017). Iris-claw intraocular lens and scleral-fixated posterior chamber intraocular lens implantations in correcting aphakia: a meta-analysis. *Investigative Ophthalmology & Visual Science*.

[9] Güell J. L., Verdaguer P., Elies D. (2014). Secondary iris-claw anterior chamber lens implantation in patients with aphakia without capsular support. *British Journal of Ophthalmology*.

[10] Chen Y., Liu Q., Xue C., Huang Z., Chen Y. (2012). Three-year follow-up of secondary anterior iris fixation of an aphakic intraocular lens to correct aphakia. *Journal of Cataract & Refractive Surgery*.

[11] Van Der Meulen I. J., Gunning F. P., Vermeulen M. G., De Smet M. D. (2004). Artisan lens implantation to correct aphakia after vitrectomy for retained nuclear lens fragments. *Journal of Cataract & Refractive Surgery*.

[12] Català-Mora J., Cuadras D., Díaz-Cascajosa J., Castany-Aregall M., Prat-Bartomeu J., García-Arumí J. (2017). Anterior iris-claw intraocular lens implantation for the management of nontraumatic ectopia lentis: long-term outcomes in a paediatric cohort. *Acta Ophthalmologica*.

[13] Manning S., Lanigan B., O’keefe M. (2016). Outcomes after lensectomy for children with marfan syndrome. *Journal of American Association for Pediatric Ophthalmology and Strabismus*.

[14] Sminia M. L., Odenthal M. T. P., Prick L. J. J. M., Cobben J. M., Mourits M. P., Völker-Dieben H. J. (2012). Long-term follow-up after bilateral artisan APHAKIA intraocular lens implantation in two children with marfan syndrome. *Journal of American Association for Pediatric Ophthalmology and Strabismus*.

[15] Sminia M. L., Odenthal M. T. P., Wenniger-Prick L. J. J. M., Gortzak-Moorstein N., Völker-Dieben H. J. (2007). Traumatic pediatric cataract: a decade of follow-up after artisan aphakia intraocular lens implantation. *Journal of American Association for Pediatric Ophthalmology and Strabismus*.

[16] Sminia M. L., Odenthal M. T. P., Gortzak-Moorstein N., Wenniger-Prick L. J. J. M., Völker-Dieben H. J. (2008). Implantation of the artisan iris reconstruction intraocular lens in 5 children with aphakia and partial aniridia caused by perforating ocular trauma. *Journal of American Association for Pediatric Ophthalmology and Strabismus*.

[17] Baykara M., Ozcetin H., Yilmaz S., Timuçin Ö. B. (2007). Posterior iris fixation of the iris-claw intraocular lens implantation through a scleral tunnel incision. *American Journal of Ophthalmology*.

[18] Gonnermann J., Torun N., Klamann M. K. J. (2013). Visual outcomes and complications following posterior iris-claw aphakic intraocular lens implantation combined with penetrating keratoplasty. *Graefe’s Archive for Clinical and Experimental Ophthalmology*.

[19] Anbari A., Lake D. B. (2015). Posteriorly enclavated iris claw intraocular lens for aphakia: long-term corneal endothelial safety study. *European Journal of Ophthalmology*.

[20] Forlini M., Soliman W., Bratu A. (2015). Long-term follow-up of retropupillary iris-claw intraocular lens implantation: a retrospective analysis. *BMC Ophthalmology*.

[21] Buhren J., Kasper T., Terzi E., Kohnen T. (2004). Higher order aberrations after implantation of an iris claw pIOL (ophtec artisan) in the phakic eye. *Ophthalmologe*.

[22] Tahzib N. G., Macrae S. M., Yoon G. (2008). Higher-order aberrations after implantation of iris-fixated rigid or foldable phakic intraocular lenses. *Journal of Cataract & Refractive Surgery*.

[23] Peris-Martínez C., Artigas J. M., Sánchez-Cortina I., Felipe A., Díez-Ajenjo A., Menezo J. L. (2009). Influence of optic quality on contrast sensitivity and visual acuity in eyes with a rigid or flexible phakic intraocular lens. *Journal of Cataract & Refractive Surgery*.

[24] Chung S.-H., Lee S. J., Lee H. K., Seo K. Y., Kim E. K. (2007). Changes in higher order aberrations and contrast sensitivity after implantation of a phakic artisan intraocular lens. *Ophthalmologica*.

[25] Wang X. G., Dong J., Wang X. L., Wu Q. (2013). IOL tilt and decentration estimation from 3 dimensional reconstruction of OCT image. *PLoS One*.

[26] Holladay J. T., Piers P. A., Koranyi G. (2002). A new intraocular lens design to reduce spherical aberration of pseudophakic eyes. *Journal of Refractive Surgery*.

[27] Piers P. A,, Weeber H. A, Artal P., Norrby S. (2007). Theoretical comparison of aberration-correcting customized and aspheric intraocular lenses. *Journal of Refractive Surgery*.

[28] Altmann G. E., Nichamin L. D., Lane S. S., Pepose J. S. (2005). Optical performance of 3 intraocular lens designs in the presence of decentration. *Journal of Cataract & Refractive Surgery*.

[29] Pérez-Vives C., Ferrer-Blasco T., Madrid-Costa D., García-Lázaro S., Montés-Micó R. (2013). Optical quality comparison of conventional and hole-visian implantable collamer lens at different degrees of decentering. *American Journal of Ophthalmology*.

[30] Pérez-Vives C., Ferrer-Blasco T., Madrid-Costa D., García-Lázaro S., Montés-Micó R. (2014). Optical quality of aspheric toric intraocular lenses at different degrees of decentering. *Graefe’s Archive for Clinical and Experimental Ophthalmology*.

[31] Baumeister M., Bühren J., Kohnen T. (2009). Tilt and decentration of spherical and aspheric intraocular lenses: effect on higher-order aberrations. *Journal of Cataract & Refractive Surgery*.

[32] Sauer T., Mester U. (2013). Tilt and decentration of an intraocular lens implanted in the ciliary sulcus after capsular bag defect during cataract surgery. *Graefe’s Archive for Clinical and Experimental Ophthalmology*.

[33] Taketani F., Matuura T., Yukawa E., Hara Y. (2004). Influence of intraocular lens tilt and decentration on wavefront aberrations. *Journal of Cataract & Refractive Surgery*.

[34] Korynta J., Bok J., Cendelin J., Michalova K. (1999). Computer modeling of visual impairment caused by intraocular lens misalignment. *Journal of Cataract & Refractive Surgery*.

[35] Atchison D. A. (1989). Optical design of intraocular lenses. III: on-axis performance in the presence of lens displacement. *Optometry and Vision Science*.

[36] Grenga P. L., Fragiotta S., Meduri A. (2013). Fixation stability measurements in patients with neovascular age-related macular degeneration treated with ranibizumab. *Canadian Journal of Ophthalmology*.

